# Neurotechnology-Based, Intensive, Supplementary Upper-Extremity Training for Inpatients With Subacute Stroke: Feasibility Study

**DOI:** 10.2196/56397

**Published:** 2025-02-13

**Authors:** Reut Binyamin-Netser, Shirley Handelzalts, Noy Goldhamer, Inbar Avni, Adi Tayer Yeshurun, Yogev Koren, Ofri Bibas Levy, Shilo Kramer, Simona Bar Haim, Lior Shmuelof

**Affiliations:** 1 Department of Cognitive and Brain Sciences, Ben-Gurion University of the Negev, 1 Ben-Gurion Ave, PO Box 653, Beer-Sheva, 8410501, Israel, 972 732113201; 2 Translational Neurorehabilitation Laboratory, Adi Negev Rehabilitation Hospital, Ofakim, Israel; 3 The Zelman Center for Brain Sciences Research, Ben-Gurion University of the Negev, Beer Sheva, Israel; 4 Department of Physical Therapy, Ben-Gurion University of the Negev, Beer Sheva, Israel; 5 Adi Negev Rehabilitation Hospital, Ofakim, Israel

**Keywords:** stroke, rehabilitation, neurotechnology

## Abstract

**Background:**

Upper-extremity hemiparesis is a common and debilitating impairment after stroke, severely restricting stroke survivors’ ability to participate in daily activities and function independently. Alarmingly, only a small percentage of stroke patients fully recover upper extremity function. Animal models indicate that high-dose upper extremity training during the early poststroke phase can significantly enhance motor recovery. However, translating such programs for human patients remains challenging due to resource limitations, patient compliance issues, and administrative constraints.

**Objective:**

This study aimed to assess the feasibility and potential efficacy of an intensive, video game–based upper-extremity training protocol designed to improve movement quality during inpatient stroke rehabilitation. Additionally, it evaluated the resources required for this intervention. Specifically, the protocol provides high-intensity, high-dose training to facilitate motor recovery by engaging patients in targeted interactive exercises.

**Methods:**

Twelve patients with upper-extremity hemiparesis completed a 4-week intensive training program comprising 40 sessions of 60 minutes; the training was conducted for 2 hours per day, 5 days per week. This was delivered in addition to standard care, which included 3 therapeutic sessions daily. Two video game–based platforms were used: one platform (tech 1) targeted proximal movements involving the shoulder and elbow, while the second platform (tech 2) emphasized distal movements of the wrist and fingers. Feasibility was assessed using the measure of time on task and measures of patients’ motivation and engagement. Potential effectiveness was assessed using the Fugl-Meyer Assessment of the upper extremity (FMA-UE) scale, Action Research Arm Test (ARAT), and Stroke Impact Scale (SIS).

**Results:**

Of the 12 patients, 8 completed the full protocol, 3 completed 34‐38 sessions, and 1 completed 27 sessions. On average, patients actively engaged in exercises for 35 (SD 4) minutes per hour on the proximal platform (tech 1) and 37 (SD 2) minutes on the distal platform (tech 2). Patients reported high motivation and enjoyment throughout the sessions, with an Intrinsic Motivation Inventory enjoyment score of 6.49 (SD 0.66) out of 7. Pain levels were minimal, with a visual analogue scale (VAS) mean score of 2.00 (SD 2.32). Significant improvements were observed in motor function assessments: the mean improvement in FMA-UE score was 16.5 (SD 10.2) points, ARAT scores increased by 22.9 (SD 13.1) points, and the SIS Hand Function and Recovery score showed a mean delta of 1.23 (SD 0.80) points and a 23.33% (SD 21.5%) improvement, respectively.

**Conclusions:**

These findings demonstrate that a high-dose, high-intensity, video game–based training protocol is feasible and can be successfully integrated into subacute stroke rehabilitation. Additionally, preliminary evidence suggests that this supplementary intervention may be effective in enhancing motor recovery. This approach holds promise for future stroke rehabilitation protocols by offering an engaging, high-dose, and high-intensity program during early recovery.

## Introduction

Stroke is the second leading cause of death and the third leading cause of disability-adjusted life-years [1]. One year after a stroke, patients have restricted participation [2]. It is estimated that one- to two-thirds of stroke survivors worldwide require some assistance or are fully dependent on caregivers for activities of daily living [3,4]. The most common impairment after stroke is hemiparesis [5]. Six months after stroke, only 12% of patients regain full functional recovery of the upper extremity (UEs) [6,7]. A poor recovery from stroke significantly impacts the lives of not only the individuals who experience the stroke, but also their families and caregivers.

Effective neurorehabilitation is essential for enhancing stroke recovery [8]. Nevertheless, at 3 months after stroke, patients achieve only 70% of their maximal potential recovery of the UEs [9-12]. One potential way to improve recovery is to increase the dosage of treatment. Increasing treatment dosage beyond standard care has been shown to be effective for the UEs [13-17]. Furthermore, the timing of treatment delivery is also important, as both nonhuman stroke models and studies in humans demonstrate a time-sensitive period of plasticity [18,19]. For example, adding 20 hours of training during the acute and subacute phases was shown to be effective in improving UE motor recovery, whereas adding the same number of training hours during the chronic phase was not [19,20]. Importantly, most studies investigating the effect of supplementary treatments were conducted with chronic stroke patients (>6 months after stroke onset) [13,14,21]. While the effect of treatment dosage on subacute recovery has been examined before in small-scale studies [15-17], its feasibility and potential efficacy needs to be established. Increased treatment dosage and intensity in the subacute phase should be defined with respect to the standard care that the patients receive. We define an increase in dosage as a supplementary treatment of more than 1 hour a day for at least 4 weeks.

The intensity and content of the training program may also affect recovery. It has been reported that patients make functional movement in only half of the therapy sessions, with 32 functional repetitions per session [22]. Furthermore, in clinical practice, rehabilitation therapies focus on the level of function (ie, task-oriented training, focusing on completing a task), rather than the level of motor impairment and quality of movement, which focus on the typicality of the performed movements [22]. Indeed, previous trials with low doses of task-oriented therapies show limited effects [23-26]. Considering these results, it was recently suggested to emphasize the quality of movement, training intensity, and an enriched environment in subacute stroke rehabilitation [27]. One way to achieve these goals is by integrating engaging video games in therapy. Adding these treatments to conventional therapy was shown to be effective (compared to conventional therapy alone [16,28]) and feasible [29].

Applying these recommendations in the subacute stroke population has several challenges. A major difficulty is that stroke patients are often withdrawn and passive, and spend extended time being sedentary [30]. Supplementary treatments should therefore be highly motivating to address the potential low compliance of the patients. Another challenge is related to the administrative complexity of adding substantial amounts of treatment to the schedule of the patients without compromising their care. A third challenge is recruiting enough personnel for supplementary treatments. Lastly, reliance on video game technologies that provide patients with online feedback on their performance requires highly reliable technologies that can be controlled by nontechnical staff. All these aspects should be developed in the context of feasibility and implementation studies.

The aim of this study was to implement and assess the feasibility of adding 40 hours of UE training based on engaging video games that emphasize the quality of movement and intensity during the early subacute phase after a stroke. We specifically aimed to (1) evaluate the feasibility of adding 2 hours a day of intensive video game–based rehabilitation during a period of 4 weeks, (2) evaluate the resources and ability to manage and implement the study and intervention, and (3) conduct a preliminary evaluation of patients’ responses to the intervention.

## Methods

### Patients

Twelve patients in the subacute phase of stroke were recruited for the study. The patients were recruited at the Adi Negev Nahalat Eran Rehabilitation Village (Israel). Inclusion criteria were as follows: age ≥18 years; ischemic or hemorrhagic stroke (hemispheric or brainstem), confirmed by computed tomography or magnetic resonance imaging; first-ever stroke or previous stroke with no UE weakness before the second incident; ≤1 week to ≤6 weeks after stroke onset, active shoulder flexion of at least 20° and partial wrist and/or finger active movement (due to a limitation of the technologies used in the intervention); Fugl-Meyer Assessment of the upper extremity (FMA-UE) score <58; and the ability to provide informed consent. Patients were excluded if they had a painful shoulder limiting an active forward reach, severe spasticity, non-neural loss of range of motion, cognitive or communication impairments as determined by the clinical team, or an unstable medical condition.

All patients were included in the final analyses. All enrolled patients had experienced a first stroke (ie, there were no cases of recurrent stroke in the population).

### Intervention

#### Overview

The aim of the program was to increase the amount of UE practice using customized video game–based platforms that included immersive, challenging, and rewarding virtual environments. In each session, patients had to achieve an explicit goal while receiving online feedback and rewards for goal attainment. The intervention was composed of 120 minutes of UE training a day (divided into 2 therapy sessions of 60 minutes), and was given for 4 weeks, for a total of 40 hours. Each session was staffed with a 1:1 physical therapist (PT)/occupational therapist (OT) to patient ratio.

We used two platforms: one for proximal arm training (shoulder and elbow; tech 1) and another for distal arm training (wrist and fingers; tech 2).

Training on both platforms was conducted while the patients were sitting on a plastic bath chair with back support and no arm support. The legs of the chair had an antislip rubber coating. On both platforms, a PT or OT was present throughout each session and provided verbal and tactile feedback to ensure movement quality and lack of compensations, and to encourage exploration of the full workspace (in tech 1). Each session was dedicated to a single platform. At least 3 sessions per week were dedicated to each platform.

Patients continued with their regular rehabilitative routine, which included daily physical and occupational therapy sessions and speech therapy if needed, as well as group work, hydrotherapy, and gym workouts during weekdays (Sunday to Friday). The regular rehabilitation protocol included at least 3 treatments a day. Patients were also encouraged to work on cardiovascular fitness during the program, as well as to use their paretic UE in daily activities. Outcome measures were collected throughout the intervention period on a session-by-session basis. To evaluate the potential effectiveness of the intervention, motor function was assessed before the intervention, immediately after the intervention, and at 12 (±14 days) and 24 (±14 days) weeks after stroke. Follow-up assessments were scheduled according to the stroke time and not the intervention to assess the potential effectiveness of the treatment at the end of the early and late subacute stages.

#### MindPod Dolphin (Tech 1) for Proximal Arm Training

**Figure 1.**
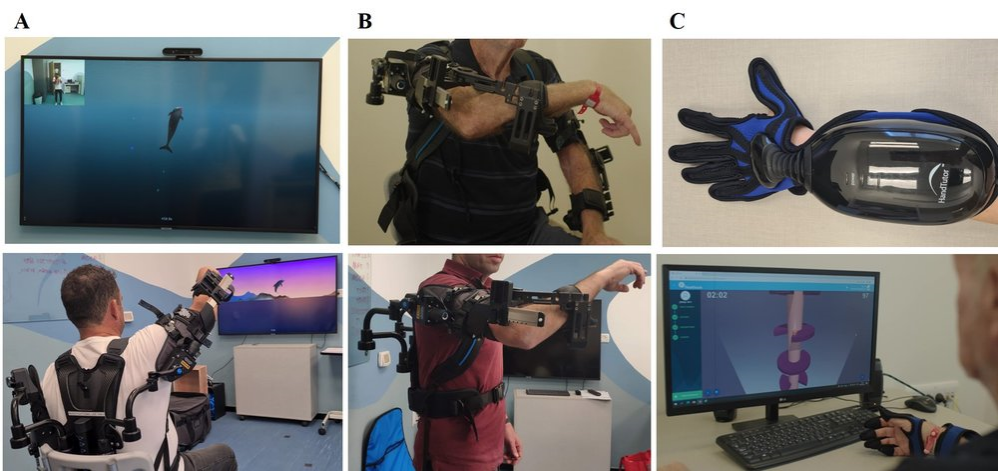
(A) Tech 1: MindPod. (B) Ekso UE vest for arm weight support. (C) Tech 2: HandTutor, an ergonomic, wearable glove.

The patients used a custom-designed, immersive, animation-based experience named MindPod Dolphin, which is based on patented technology licensed from KATA at Johns Hopkins University to MSquare Healthcare Inc, a MindMaze Group company (Figure 1A) [16]. In this game, patients control the swimming of a virtual dolphin by moving their shoulders and elbow joints and performing complex exploratory movements. The dolphin swims through different ocean scenes with multiple goals such as chasing and eating fish, escaping a shark, and jumping out of the water. This game was designed to promote the movement quality of the shoulder and elbow in all planes (3D movements; this included shoulder abduction, adduction, flexion, and extension and elbow flexion and extension) throughout the active ranges of motion. The game uses a real-time motion capture system. The paretic arm was supported by a passive mechanical exoskeleton vest (Ekso UE; Ekso Bionics; Figure 1B). The degree of support was provided by the exoskeleton using springs with different tension levels. The amount of spring tension needed was adjusted for each patient to allow active movement of the shoulder to at least 90° of flexion. The support of the vest allowed the patients to play with their paretic arm in an extended active range in all directions. The arm weight support level was titrated as the patient progressed through the sessions by reducing the level of support or removing the vest. No active assistance was given by the therapist during the intervention. The game was designed in a way that every level was more difficult than the one before. Each level had a goal (eg, to eat a specific number of fish), and when the goal was reached, the following level began.

#### HandTutor (Tech 2) for Distal Arm Training

The HandTutor system (MediTouch; Figure 1C) consists of an ergonomic wearable glove and a dedicated video game that allow practice of active wrist movements, grip control, and finger individuation in a virtual environment. Patients progress through games while adjusting the range of motion that is being practiced according to their abilities. Patients perform flexion and extension movements of their fingers or wrist separately or simultaneously. In each session, the therapist chooses the trained movements (emphasizing grip abilities and finger individuation). In one game, for example, patients controlled a fishing rod up and down by flexing and extending an individual finger to catch swimming fish. The finger range of movement was set before the game started. If the task was too easy, the therapist increased the difficulty level by making the fish move faster or by adding another control dimension, such as adding movement of the wrist on top of the fingers in a manner that the fingers controlled the fishing rod movement (up or down) and the wrist controlled the boat movement (left or right).

### Outcome Measures of Feasibility

Adherence rates to study procedures were documented by the therapists for each session. Time on task (ToT) was measured in each session (in minutes) using a stopwatch. The distance that the arm reached on the task (in meters) was measured in tech 1. Game levels and the amount of weight support were documented by the therapist in each session, as were rehabilitation sessions outside of the intervention (ie, usual care) during the study period. Pain level was monitored using the visual analogue scale (VAS) at the end of each session and before and after the entire intervention. The VAS is a self-report measure consisting of a 10-cm line with a statement at each end representing one extreme of pain intensity (“no pain” and “pain as bad as it could possibly be”). The patient marks the line with a pen at a point corresponding to their present pain level [31]. Exercise intensity was measured using the rating of perceived exertion (RPE), where patients subjectively rate their level of exertion during exercise (1=“did not put in an effort at all” to 10=“put in an extreme effort”) [32]. Participation was measured using the Pittsburgh Rehabilitation Participation Scale (PRPS), a clinician-rated instrument to assess a patient’s participation in therapy using effort and motivation estimates [33]. This measure was taken at the end of each practice session. Motivation was measured using the Intrinsic Motivation Inventory (IMI; Multimedia Appendix 1), a multidimensional measurement intended to assess patients’ subjective experience related to a target activity in laboratory experiments. The instrument assesses patients’ interest/enjoyment, perceived competence, effort, value/usefulness, felt pressure and tension, and perceived choice while performing a given activity. We used the Task Evaluation Questionnaire, a specific version of the IMI, to assess only 4 subscales: interest/enjoyment, perceived competence, pressure/tension, and perceived choice. Motivation was assessed through rating the agreement with proposed statements using a Likert scale (1=“not at all true” to 7=“very true”) [34]. Motivation was assessed at the end of the intervention period.

Adverse events and problems with the operation of the interventions were documented by the therapists throughout the study period.

We found that 4.2% of data were missing from the RPE reports, 5% from the PRPS reports, and 9% from the VAS reports. The data were missing due to the failure of therapists to fill in the forms. Statistical analyses were conducted without the missing values.

### Outcome Measures of Potential Efficacy

The FMA-UE assessment was used to assess motor impairment. The FMA-UE is scored on an ordinal 3-point scale. The maximum score for the FMA-UE is 66 for each arm, with a higher score indicating better arm motor status [35]. The FMA-UE has shown good reliability, validity, and sensitivity to poststroke motor changes [36]. The Action Research Arm Test (ARAT) was used to assess motor activity using 19 tests of arm motor function, both distally and proximally (grasp, grip, pinch, and gross movement). Each test is given an ordinal score of 4 values, with higher values indicating better arm motor status. The total ARAT score is the sum of the 19 tests, and thus the maximum score is 57 [37]. The ARAT demonstrates high reliability and good validity, as well as sensitivity to spontaneous and therapy-related gains after a stroke [38] The Stroke Impact Scale (SIS) hand domain (section 7; Multimedia Appendix 2), version 2.0, was used to assess disability and health-related quality of life using a questionnaire. It includes 64 self-report items, divided into 8 areas: strength (physical difficulties), memory and thought, emotion, communication, activities of daily living, mobility, manual function, and social participation. The rating of the items was carried out on an ordinal scale of 5 levels. A separate score was given to each field according to an algorithm. A final score was given in the range between 0 and 100, where lower scores indicate a negative effect in that area on health and perception of quality of life. In addition, overall recovery from the event (section 9) was assessed by a percentage rating on a VAS between 0 (no recovery) and 100 (full recovery) [39,40].

### Statistical Analyses

All analyses and statistical calculations were performed using SPSS (version 21; IBM Corp). Means and SDs were calculated for all outcome measures. Paired-sample 2-tailed *t* tests were performed to compare the motor outcome measures before and after the intervention.

### Ethical Considerations

The study was approved by the Regional Ethical Review Board at Sheba Medical Center, Israel (6218‐19-SMC), and registered with ClinicalTrials.gov (NCT04737395) prior to the start of enrollment. All patients signed a consent form before participating in the experiment. The original informed consent form allowed secondary analyses without additional consent. All study data are anonymous. The enrolled patients volunteered for this study.

## Results

### Overview

Twelve patients participated in the study, aged 44 to 71 years (mean age 61.67, SD 8.80 years; n=3 women; mean time after stroke 32.42, SD 16.45 days). Patients varied in the amount of formal education they had received (0-15 years), and their medical conditions (these included obesity, schizophrenic disorder, hyperlipidemia, hypothyroidism, essential hypertension, and depression) (Table 1).

**Table 1.** Patients’ characteristics (N=12).

Patient	Age(years)	Sex	Education(years)	Time from stroke to intervention (days)	Type of stroke	Dominant hand	Impaired side	Stroke location	MoCA^a^ score
1	49	M^b^	12	38	H^c^	L^d^	R^e^	Thalamus and basal ganglia (L)	20
2	64	M	12	27	I^f^	R	R	Thalamus (L)	25
3	44	M	14	69	H	R	R	Basal ganglia (L)	19
4	66	M	8	49	I	L	R	MCA^g^ (L)	18
5	56	F^h^	12	25	H	R	R	Basal ganglia (L)	25
6	68	F	10	44	I	R	L	Basal ganglia (R)	14
7	71	M	0	34	I	R	R	Occipital (L)	8
8	55	F	12	25	I	L	L	MCA (R)	21
9	61	M	15	15	I	R	L	Periventricular (R)	20
10	69	M	10	10	I	R	L	Periventricula (R)	19
11	70	M	12	17	I	R	R	PCA^i^ (L)	22
12	67	M	12	36	I	R	R	Periventricular (L)	27
Overall, mean (SD)	61.7 (8.8)	—^j^	10.7 (3.8)	32.4 (16.4)	—	—	—	—	19.8 (4.9)

a MoCA: Montreal Cognitive Assessment score (maximum score=30); MoCA score was converted from Mini Mental State Examination score.

b M: male.

c H: hemorrhagic stroke.

d L: left.

e R: right.

f I: ischemic stroke.

g MCA: middle cerebral artery.

h F: female.

i PCA: posterior crebral artery.

j Not applicable.

### Training Adherence and ToT

**Figure 2.**
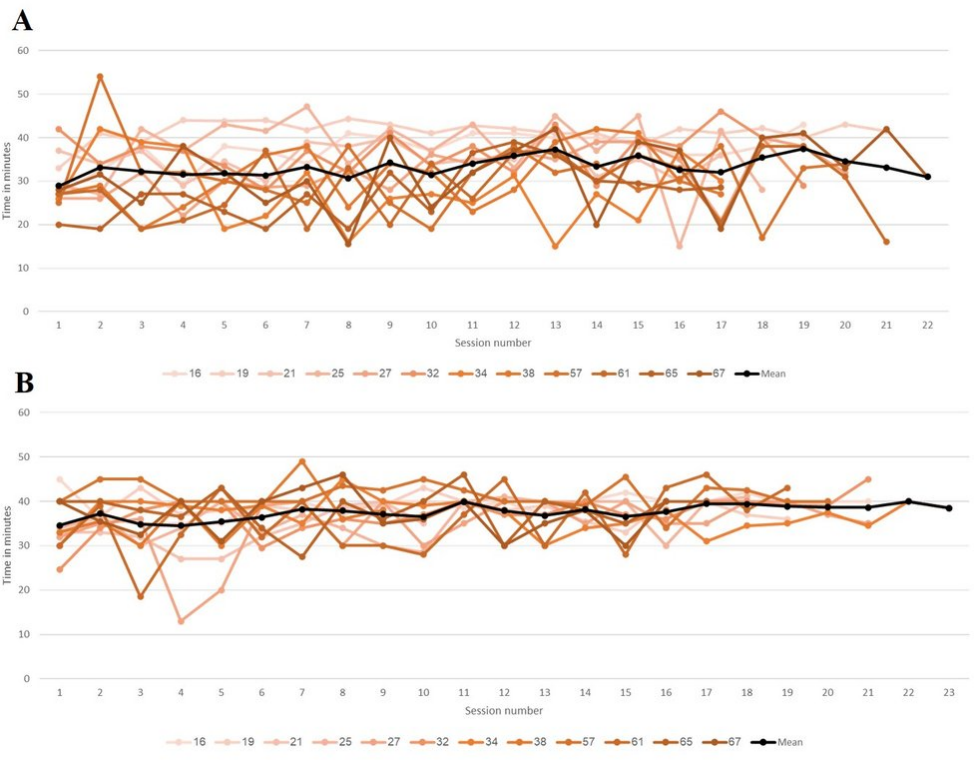
Total time on task. (A) Tech 1 (shoulder and elbow movement practice). (B) Tech 2 (wrist and finger movement practice). The data represent each session. All 12 patients are represented. Each patient is represented by a different line. The average of all patients is represented by the black lines.

**Figure 3.**
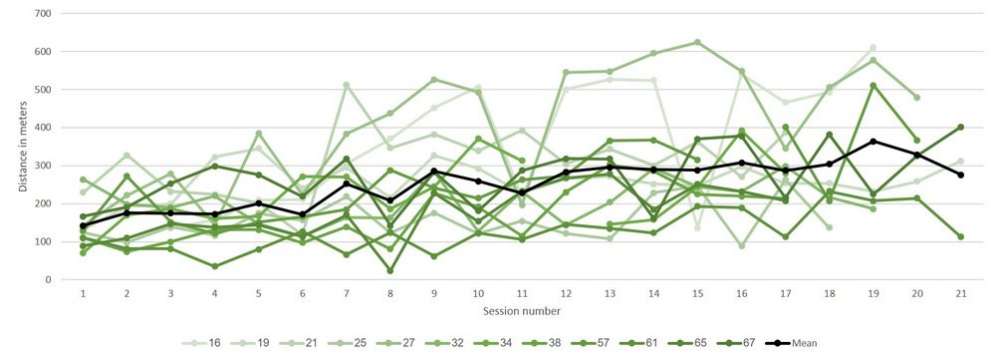
Total arm distance for tech 1 (shoulder and elbow movement practice) for each of the sessions. All 12 patients are represented. Each patient is represented by a different line. The average of all patients is represented by the black line.

The mean number of delivered sessions was 38 (SD 4). A total of 8 of our 12 (67%) patients completed the 40 intervention sessions. Patients 3 and 11 did not complete the protocol due to COVID-19, patient 4 due to poor motivation at the end of the intervention, and patient 12 due to a medical condition (Table 2). All patients completed pre- and postintervention assessments. A total of 9 (75%) patients completed the 12-week postintervention assessment, and 7 (58%) completed the 24-week postintervention assessment (the missing data is because the patients failed to attend). The mean ToT was 35 (SD 2.44) minutes per 1-hour session (33, SD 4.35 minutes for tech 1 and 37, SD 2.09 minutes for tech 2; Figure 2). For tech 1, total arm distance was also measured, reaching a mean distance of 246 (SD 87.35) meters (Figure 3). In addition to the intervention, all patients received an average of 2.7 (SD 0.36) standard care rehabilitation hours a day. In total, patients received an average of 54 (SD 8.05) hours of standard care rehabilitation during the intervention period.

**Table 2.** Training attendance and time on task (ToT).

Patient	Total intervention sessions	Mean ToTtech 1 (minutes), mean (SD)	Mean ToTtech 2 (minutes), mean (SD)
1	40	37 (4)	39 (2)
2	40	41 (2)	38 (3)
3	27	35 (4)	33 (4)
4	36	37 (8)	36 (4)
5	41	32 (6)	34 (7)
6	40	36 (5)	37 (4)
7	40	28 (8)	37 (3)
8	40	31 (7)	37 (6)
9	40	31 (8)	41 (3)
10	40	29 (7)	35 (7)
11	34	27 (6)	37 (4)
12	38	31 (8)	37 (5)
Overall, mean (SD)	38 (4)	33 (4)	37 (2)

The effort level that was reported by the patients was high (RPE; see Methods section). The mean effort patients reported was 7.14 (SD 2.18, on a scale of 10) points (for tech 1: 7.38, SD 2.21 points; for tech 2: 6.90, SD 2.21 points). This suggests that patients were motivated to make an effort in each training session. Participation assessment by the therapists was also high (PRPS; see Methods section). The mean participation of patients was 5.42 (SD 0.49) points (for tech 1: 5.45, SD 0.49 points; for tech 2: 5.40, SD 0.51 points). Pain was measured using the VAS (see Methods section). This measure was taken after each session to determine if the training was associated with any pain. The mean VAS patients reported was 2.00 (SD 2.32) points (for tech 1: 2.39, SD 2.73 points; for tech 2: 1.61, SD 1.86 points). This suggests that pain was generally low.

Motivation was assessed at the end of the intervention (IMI; see Methods section). The mean score in each category was as follows: interest/enjoyment was 6.49 (SD 0.66), perceived competence was 6.18 (SD 0.80), perceived choice was 5.42 (SD 1.39), and pressure/tension was 1.75 (SD 0.97) points. This suggests that patients enjoyed the intervention, felt that they could do it, and felt that joining the intervention was their choice. They did not feel under pressure during the intervention sessions.

No adverse events were recorded during the intervention sessions.

### Feasibility of Operation

The intervention hours were scheduled before and after the regular rehabilitation hours, from 7 to 8 AM and from 3 to 4 PM, to minimize the effect of the intervention on standard care (3 treatments per day) and to secure therapists for the intervention. This decision led to extension of the treatment day for the enrolled patients, and was dependent on good cooperation between the ward management staff, who had to make sure the patients were prepared to start the rehabilitation day earlier. Another challenge raised by this decision was that even though the interventions were conducted on a single patient at a time, 7 trained therapists were required to fill the intervention schedule.

### Motor Outcomes

The scores of the outcome measures before and after intervention are shown for each patient in Table 3. The average FMA-UE recovery was 16.50 (SD 10.2) points. This difference is significant (*t*_11_=−5.58; *P*<.001) and higher than the minimal clinically important difference (MCID; 9‐10 points) [41]. The average ARAT recovery was 22.92 (SD 13.1) points. This difference is also significant (*t*_11_=−6.05; *P*<.001) and higher than the MCID (12‐17 points) [42]. The average SIS Function and SIS Recovery changes were 1.23 (SD 0.8) points and 23.33% (SD 21.5%), respectively. These differences were significant (*t*_11_=−5.01*; P*<.001 and *t*_11_=−3.77; *P*=.003, respectively). The outcome measures that were obtained at the end of the intervention were largely stable at 12 and 24 weeks after stroke: the average FMA-UE was 48.78 (SD 12.08) and 48.57 (SD 11.31) points, respectively (in comparison to the average of 49.75, SD 10.87 points after the intervention). The average ARAT scores were 39.67 (SD 20.12) and 35.86 (SD 18.08) points, respectively (in comparison to the average of 39.0, SD 16.89 points after the intervention). The average SIS Function scores were 3.44 (SD 0.73) and 3.11 points (SD 1.26), respectively (in comparison to the average of 2.98, SD 1.01 points after the intervention). The average SIS Recovery scores were 69.44% (SD 17.04%) and 72.14% (SD 14.39%), respectively (in comparison to the average of 71.25%, SD 13.84% after the intervention). Generally, patients maintained their level of motor function 12 and 24 weeks after stroke.

**Table 3.** Stroke patients’ measurement scores.

Patient	Preintervention FMA-UE^a^ motor score	FMA-UE motor change^b^	Preintervention ARAT^c^ score	ARAT score change	Preintervention SIS^d^ Function score	SIS Function change	Preintervention SIS Recovery (%)	SIS Recovery change
1	41	21	24	32	2.6	1	30	40
2	27	9	2	10	1	0.8	20	60
3	25	3	5	0	2.4	0.2	85	-5
4	18	26	3	29	1	0.2	45	25
5	25	28	0	49	1.2	1.2	10	40
6	40	12	36	18	2	2.2	50	10
7	33	10	26	14	1.4	1	30	20
8	38	8	12	19	2.6	1.8	80	20
9	36	27	30	27	1.4	1.4	15	55
10	30	32	11	36	1.2	3.2	80	0
11	54	3	36	14	2.4	0.6	60	15
12	32	19	8	27	1.8	1.2	70	0
Overall, mean (SD)	33.3 (9.5)	16.5 (10.2)	16.1 (13.5)	22.9 (13.1)	1.7 (0.6)	1.2 (0.8)	47.9 (27.0)	23.3 (21.5)

a FMA-UE: Fugl-Meyer Assessment of the upper extremity motor score (maximum score=66).

b Change indicates the delta between the scores before and after the intervention.

c ARAT: Action Research Arm Test (maximum score=57).

d SIS: Stroke Impact Scale (this scale has 2 subscales: Function, maximum score=5, and Recovery [in %], maximum=100%).

## Discussion

### Principal Findings

This study demonstrates the feasibility of an intensive supplementary subacute training protocol of 40 sessions. In most cases, the protocol was implemented successfully, and incomplete implementation was due to reasons that were unrelated to the intervention protocol. The proportion of ToT to total time was above 60%, and participants found the intervention to be motivating, enjoyable, and engaging.

### Intensive UE Training in Patients With Subacute Stroke Is Feasible

We found that it is possible to add 2 hours of intensive UE training per day during hospitalization. This finding has 2 aspects: patients in the subacute stage could endure 2 additional hours of intensive training a day and the medical system could deliver the training. The following elements are recommended for program success: (1) administrative support—this program required the full cooperation of the rehabilitation ward staff to prepare the patients on time (prioritizing them) and to organize their treatment schedule in a way that considered factors such as meals, rest periods, and the mandatory number of usual care treatments per day; (2) engagement of therapists and patients—multiple therapists should be trained to administer the intervention to ensure a smooth flow of treatments (without cancelations; coordination of the clinical staff is also important for information exchange regarding the participating patients); (3) reliable and attractive technologies—the technologies we chose to use in this intervention were reliable, highly motivating, enjoyable, and challenging.

### Feasibility Outcome Measures

We expected to reach two-thirds of the total time dedicated for each training session in ToT (ie, 40 minutes ToT per 60-minute session), which would have been similar to the expected ratio in a standard therapy session. However, we reached only 35‐37 minutes per hour due to extended preparation time, technology maintenance, and patient fatigue. Integrating the intervention within the standard care schedule may increase the proportion of ToT. Notably, compared to other studies that investigated intervention protocols in the subacute phase after a stroke, our protocol is very intensive [19,20,23-26,43]. Importantly, in addition to the potential benefits of early intervention [19,20,29], the ability to provide an intensive intervention protocol in the subacute phase during hospitalization is highly cost-effective since it does not require inpatient or outpatient programs, such as those that are used in the chronic phase after a stroke [13,14].

### The Motor Benefits of the Intervention

Substantial improvement was reported in all motor outcome measures. This improvement was larger compared to other reported recovery measures [13,14,44]. A new study showed that adding 30 hours of UE training resulted in better motor improvement than the standard of care alone [16]. That study showed an improvement of 12.5‐13.4 points in the FMA-UE (in comparison to 16.5 points of improvement in our study) and 13.4‐14.7 points in the ARAT (in comparison to 22.9 points of improvement in our study). Based on the apparent increased recovery following 40 hours of training, we recommend studying the efficacy of a supplementary intervention protocol of at least 40 hours. Furthermore, the effect of supplemental treatment on recovery may not be independent of standard care treatments. We therefore call for reporting the standard care content and dosage in future intervention studies.

### Implementation Recommendations

Implementation of the intensive treatment could be improved in different ways: the efficacy and feasibility of the intervention could be enhanced by increasing the number of patients per therapist (OT or PT), so that multiple patients can practice at the same time. Practice could also be administered by a PT or OT assistant. Moreover, it may be possible to develop an implementation model where treatment is given during the rehabilitation day and not off hours. Other suggested avenues for improvement are to expand this protocol in time and to include a telerehabilitation component. Although this type of rehabilitation is relatively new and has not been extensively studied [45], it has been shown to be feasible [44]. Therefore, we suggest further studies regarding the implementation of high-dosage and high-intensity treatment with a such a component. Ultimately, the efficacy of this intervention compared to the standard of care and to an increased dosage of the standard of care should be studied in randomized controlled trials.

### Limitations

Since this is a feasibility study, the reported recovery measures cannot be assigned to the intervention. A randomized controlled trial should be conducted to establish the efficacy of the intervention. Another limitation is the lack of knowledge regarding the content and intensity of standard UE training that our patients received. We can estimate that out of the 3 hours of standard care treatment they received a day, about 1.5 hours were dedicated to UE training and the other 1.5 were dedicated to other areas, such as cognition, the lower extremities, and psychological aspects. We recommend that future studies monitor the content of standard care. Last, as treatment intensity and dosage increases, fatigue, a prominent effect of stroke [46], may become a limiting factor of training adherence. We suggest that future studies assess fatigue using a valid and reliable tool such as the Fatigue Severity Scale [47].

### Conclusion

Intensive UE training is practical and well received by patients with subacute stroke. Successful implementation depends on the clinical setting, technologies, and resources. We call for studying the implementation of this protocol with multiple patients at a time and during clinical days and for randomized control trials to study the efficacy of the intervention. Our preliminary results suggest that subacute intensive rehabilitation may improve motor recovery after a stroke.

## Supplementary material

10.2196/56397 Multimedia Appendix 1 Intrinsic Motivation Inventory.

10.2196/56397 Multimedia Appendix 2 Stroke Impact Scale.

## Abbreviations

ARAT: Action Research Arm Test
FMA-UE: Fugl-Meyer Assessment of the upper extremity
IMI: Intrinsic Motivation Inventory
MCID: minimal clinically important difference
OT: occupational therapist
PRPS: Pittsburgh Rehabilitation Participation Scale
PT: physical therapist
RPE: rating of perceived exertion
SIS: Stroke Impact Scale
ToT: time on task
UE: upper extremity
VAS: visual analog scale
