# Supplementary material for: Neurotechnology-Based, Intensive, Supplementary Upper-Extremity Training for Inpatients With Subacute Stroke: Feasibility Study
Source: JMIR Serious Games. 2025 Feb 13;13:e56397. doi: 10.2196/56397 (PMC11841746; doi:10.2196/56397)
Supplement: Multimedia Appendix 1 [file games-v13-e56397-s001.pdf]

Intrinsic Motivation Inventory (IMI)

Scale Description

The Intrinsic Motivation Inventory (IMI) is a multidimensional measurement device intended to assess participantsÕ subjective experience related to a target activity in laboratory experiments. It has been used in several experiments related to intrinsic motivation and self-regulation (e.g., Ryan, 1982; Ryan, Mims & Koestner, 1983; Plant & Ryan, 1985; Ryan, Connell, & Plant, 1990; Ryan, Koestner & Deci, 1991; Deci, Eghrari, Patrick, & Leone, 1994). The instrument assesses participantsÕ interest/enjoyment, perceived competence, effort, value/usefulness, felt pressure and tension, and perceived choice while performing a given activity, thus yielding six subscale scores. Recently, a seventh subscale has been added to tap the experiences of relatedness, although the validity of this subscale has yet to be established. The **interest/enjoyment subscale is considered the self-report measure of intrinsic motivation**; thus, although the overall questionnaire is called the Intrinsic Motivation Inventory, it is only the one subscale that assesses intrinsic motivation, per se. As a result, the interest/enjoyment subscale often has more items on it that do the other subscales. The perceived choice and perceived competence concepts are theorized to be positive predictors of both self-report and behavioral measures of intrinsic motivation, and pressure/tension is theorized to be a negative predictor of intrinsic motivation. Effort is a separate variable that is relevant to some motivation questions, so is used it its relevant. The value/usefulness subscale is used in internalization studies (e.g., Deci et al, 1994), the idea being that people internalize and become self-regulating with respect to activities that they experience as useful or valuable for themselves. Finally, the relatedness subscale is used in studies having to do with interpersonal interactions, friendship formation, and so on.

The IMI consists of varied numbers of items from these subscales, all of which have been shown to be factor analytically coherent and stable across a variety of tasks, conditions, and settings. The general criteria for inclusion of items on subscales have been a factor loading of at least 0.6 on the appropriate subscale, and no cross loadings above 0.4. Typically, loadings substantially exceed these criteria. Nonetheless, we recommend that investigators perform their own factor analyses on new data sets. Past research suggests that order effects of item presentation appear to be negligible, and the inclusion or exclusion of specific subscales appears to have no impact on the others. Thus, it is rare that all items have been used in a particular experiment. Instead, experimenters have chosen the subscales that are relevant to the issues they are exploring.

The IMI items have often been modified slightly to fit specific activities. Thus, for example, an item such as ÒI tried very hard to do well at this activityÓ can be changed to ÒI tried very hard to do well on these puzzlesÓ or Ò...in learning this materialÓ without effecting its reliability or validity. As one can readily tell, there is nothing subtle about these items; they are quite face-valid. However, in part, because of their straightforward nature, caution is needed in interpretation. We have found, for example, that correlations between self-reports of effort or interest and behavioral indices of these dimensions are quite modest--often around 0.4. Like other self-report measures, there is always the need to appropriately interpret how and why participants report as they do. Ego-involvements, self-presentation styles, reactance, and other psychological dynamics must be considered. For example, in a study by Ryan, Koestner, and Deci (1991), we found that when participants were ego involved, the engaged in pressured persistence during a free choice period and this behavior did not correlate with the

self-reports of interest/enjoyment. In fact, we concluded that to be confident in oneÕs assessment of intrinsic motivation, one needs to find that the free-choice behavior and the self-reports of interest/enjoyment are significantly correlated.

Another issue is that of redundancy. Items within the subscales overlap considerably, although randomizing their presentation makes this less salient to most participants. Nonetheless, shorter versions have been used and been found to be quite reliable. The incremental R for every item above 4 for any given factor is quite small. Still, it is very important to recognize that multiple item subscales consistently outperform single items for obvious reasons, and they have better external validity.

On The Scale page, there are five sections. First, the full 45 items that make up the 7 subscales are shown, along with information on constructing your own IMI and scoring it. Then, there are four specific versions of the IMI that have been used in past studies. This should give you a sense of the different ways it has been used. These have different numbers of items and different numbers of subscales, and they concern different activities. First, there is a standard, 22-item version that has been used in several studies, with four subscales: interest/enjoyment, perceived competence, perceived choice, and pressure/tension. Second, there is a short 9-item version concerned with the activity of reading some text material; it has three subscales: interest/enjoyment, perceived competence, and pressure/tension. Then, there is the 25-item version that was used in the internalization study, including the three subscales of value/usefulness, interest/enjoyment, and perceived choice. Finally, there is a 29-item version of the interpersonal relatedness questionnaire that has five subscales: relatedness, interest/enjoyment, perceived choice, pressure/tension, and effort.

Finally, McAuley, Duncan, and Tammen (1987) did a study to examine the validity of the IMI and found strong support for its validity.

References

Deci, E. L., Eghrari, H., Patrick, B. C., & Leone, D. (1994). Facilitating internalization: The self-determination theory perspective. *Journal of Personality*, *62*, 119-142.

McAuley, E., Duncan, T., & Tammen, V. V. (1987). Psychometric properties of the Intrinsic Motivation Inventory in a competitive sport setting: A confirmatory factor analysis. *Research Quarterly for Exercise and Sport, 60,* 48-58.

Plant, R. W., & Ryan, R. M. (1985). Intrinsic motivation and the effects of self-consciousness, self-awareness, and ego-involvement: An investigation of internally-controlling styles. *Journal of Personality*, *53*, 435-449.

Ryan, R. M. (1982). Control and information in the intrapersonal sphere: An extension of cognitive evaluation theory. *Journal of Personality and Social Psychology*, *43*, 450-461.

Ryan, R. M., Connell, J. P., & Plant, R. W. (1990). Emotions in non-directed text learning. *Learning and Individual Differences*, *2*, 1-17.

Ryan, R. M., Koestner, R., & Deci, E. L. (1991). Varied forms of persistence: When free-choice behavior is not intrinsically motivated. *Motivation and Emotion*, *15*, 185-205.

Ryan, R. M., Mims, V., & Koestner, R. (1983). Relation of reward contingency and interpersonal context to intrinsic motivation: A review and test using cognitive evaluation theory. *Journal of Personality and Social Psychology*, *45*, 736-750.

The Scales

THE POST-EXPERIMENTAL INTRINSIC MOTIVATION INVENTORY
(Below are listed all 45 items that can be used depending on which are needed.)

For each of the following statements, please indicate how true it is for you, using the following scale:

1 2 3 4 5 6 7

not at all true — somewhat true — very true

Interest/Enjoyment

I enjoyed doing this activity very much
This activity was fun to do.
I thought this was a boring activity. (R)
This activity did not hold my attention at all. (R)
I would describe this activity as very interesting.
I thought this activity was quite enjoyable.
While I was doing this activity, I was thinking about how much I enjoyed it.

Perceived Competence
I think I am pretty good at this activity.
I think I did pretty well at this activity, compared to other students.
After working at this activity for awhile, I felt pretty competent.
I am satisfied with my performance at this task.
I was pretty skilled at this activity.
This was an activity that I couldnÕt do very well. (R)

Effort/Importance
I put a lot of effort into this.
I didnÕt try very hard to do well at this activity. (R)

I tried very hard on this activity.
It was important to me to do well at this task.
I didnÕt put much energy into this. (R)

Pressure/Tension
I did not feel nervous at all while doing this. (R)
I felt very tense while doing this activity.
I was very relaxed in doing these. (R)
I was anxious while working on this task.
I felt pressured while doing these.

Perceived Choice
I believe I had some choice about doing this activity.
I felt like it was not my own choice to do this task. (R)
I didnÕt really have a choice about doing this task. (R)
I felt like I had to do this. (R)
I did this activity because I had no choice. (R)
I did this activity because I wanted to.
I did this activity because I had to. (R)

Value/Usefulness
I believe this activity could be of some value to me.
I think that doing this activity is useful for ______________________
I think this is important to do because it can ______________________
I would be willing to do this again because it has some value to me.
I think doing this activity could help me to _____________________
I believe doing this activity could be beneficial to me.
I think this is an important activity.

Relatedness
I felt really distant to this person. (R)
I really doubt that this person and I would ever be friends. (R)
I felt like I could really trust this person.
IÕd like a chance to interact with this person more often.
IÕd really prefer not to interact with this person in the future. (R)
I donÕt feel like I could really trust this person. (R)
It is likely that this person and I could become friends if we interacted a lot.
I feel close to this person.

Constructing the IMI for your study. First, decide which of the variables (factors) you want to use, based on what theoretical questions you are addressing. Then, use the items from those factors, randomly ordered. If you use the value/usefulness items, you will need to complete the three items as appropriate. In other words, if you were studying whether the person believes an activity is useful for improving concentration, or becoming a

better basketball player, or whatever, then fill in the blanks with that information. If you do not want to refer to a particular outcome, then just truncate the items with its being useful, helpful, or important.

Scoring information for the IMI. To score this instrument, you must first reverse score the items for which an (R) is shown after them. To do that, subtract the item response from 8, and use the resulting number as the item score. Then, calculate subscale scores by averaging across all of the items on that subscale. The subscale scores are then used in the analyses of relevant questions.

* * * * * * * * * * * *

The following is a 22 item version of the scale that has been used in some lab studies on intrinsic motivation. It has four subscales: interest/enjoyment, perceived choice, perceived competence, and pressure/tension. The interest/enjoyment subscale is considered the self-report measure of intrinsic motivation; perceived choice and perceived competence are theorized to be positive predictors of both self-report and behavioral measures of intrinsic motivation. Pressure tension is theorized to be a negative predictor of intrinsic motivation. Scoring information is presented after the questionnaire itself.

TASK EVALUATION QUESTIONNAIRE

For each of the following statements, please indicate how true it is for you, using the following scale:

1	2	3	4	5	6	7
not at all true			somewhat true			very true

1. While I was working on the task I was thinking about how much I enjoyed it.

2. I did not feel at all nervous about doing the task.

3. I felt that it was my choice to do the task.

4. I think I am pretty good at this task.

5. I found the task very interesting.

6. I felt tense while doing the task.

7. I think I did pretty well at this activity, compared to other students.

8. Doing the task was fun.

9. I felt relaxed while doing the task.

10. I enjoyed doing the task very much.

11. I didnÕt really have a choice about doing the task.

12. I am satisfied with my performance at this task.

13. I was anxious while doing the task.

14. I thought the task was very boring.

15. I felt like I was doing what I wanted to do while I was working on the task.

16. I felt pretty skilled at this task.

17. I thought the task was very interesting.

18. I felt pressured while doing the task.

19. I felt like I had to do the task.

20. I would describe the task as very enjoyable.

21. I did the task because I had no choice.

22. After working at this task for awhile, I felt pretty competent.

Scoring information. Begin by reverse scoring items # 2, 9, 11, 14, 19, 21. In other words, subtract the item response from 8, and use the result as the item score for that item. This way, a higher score will indicate more of the concept described in the subscale name. Thus, a higher score on pressure/tension means the person felt more pressured and tense; a higher score on perceived competence means the person felt more competent; and so on. Then calculate subscale scores by averaging the items scores for the items on each subscale. They are as follows. The (R) after an item number is just a reminder that the item score is the reverse of the participantÕs response on that item.

Interest/enjoyment: 1, 5, 8, 10, 14(R), 17, 20
Perceived competence: 4, 7, 12, 16, 22
Perceived choice: 3, 11(R), 15, 19(R), 21(R)
Pressure/tension: 2(R), 6, 9(R), 13, 18

The subscale scores can then be used as dependent variables, predictors, or mediators, depending on the research questions being addressed.

* * * * * * * * * * * *

TEXT MATERIAL QUESTIONNAIRE I

For each of the following statements, please indicate how true it is for your, using the following scale as a guide:

1 2 3 4 5 6 7

not at all true	somewhat true	very true

1. While I was reading this material, I was thinking about how much I enjoyed it.
2. I did not feel at all nervous while reading.
3. This material did not hold my attention at all.
4. I think I understood this material pretty well.
5. I would describe this material as very interesting.
6. I think I understood this material very well, compared to other students.
7. I enjoyed reading this material very much.
8. I felt very tense while reading this material.
9. This material was fun to read.

Scoring information. Begin by reverse scoring items # 2 and 3. In other words, subtract the item response from 8, and use the result as the item score for that item. This way, a higher score will indicate more of the

concept described in the subscale name. Then calculate subscale scores by averaging the items scores for the items on each subscale. They are shown below. The (R) after an item number is just a reminder that the item score is the reverse of the participantÕs response on that item.

Interest/enjoyment: 1, 3(R), 5, 7, 9
Perceived competence: 4, 6,
Pressure/tension: 2(R), 8

* * * * * * * * * * * *

The next version of the questionnaire was used for a study of internalization with an uninteresting computer task (Deci et al., 1994).

ACTIVITY PERCEPTION QUESTIONNAIRE

The following items concern your experience with the task. Please answer all items. For each item, please indicate how true the statement is for you, using the following scale as a guide:

1	2	3	4	5	6	7
not at all true			somewhat true			very true

1. I believe that doing this activity could be of some value for me.

2. I believe I had some choice about doing this activity.

3. While I was doing this activity, I was thinking about how much I enjoyed it.

4. I believe that doing this activity is useful for improved concentration.

5. This activity was fun to do.

6. I think this activity is important for my improvement.

7. I enjoyed doing this activity very much.

8. I really did not have a choice about doing this activity.

9. I did this activity because I wanted to.

10. I think this is an important activity.

11. I felt like I was enjoying the activity while I was doing it.

12. I thought this was a very boring activity.

13. It is possible that this activity could improve my studying habits.

14. I felt like I had no choice but to do this activity.

15. I thought this was a very interesting activity.

16. I am willing to do this activity again because I think it is somewhat useful.

17. I would describe this activity as very enjoyable.

18. I felt like I had to do this activity.

19. I believe doing this activity could be somewhat beneficial for me.

20. I did this activity because I had to.

21. I believe doing this activity could help me do better in school.

22. While doing this activity I felt like I had a choice.

23. I would describe this activity as very fun.

24. I felt like it was not my own choice to do this activity.

25. I would be willing to do this activity again because it has some value for me.

Scoring information. Begin by reverse scoring items # 8, 12, 14, 18, 20, and 24 by subtracting the item response from 8 and using the result as the item score for that item. Then calculate subscale scores by averaging the items scores for the items on each subscale. They are shown below. The (R) after an item number is just a reminder that the item score is the reverse of the participantÕs response on that item.

Interest/enjoyment: 3, 5, 7, 11, 12(R), 15, 17, 23
Value/usefulness: 1, 4, 6, 10, 13, 16, 19, 21, 25
Perceived choice: 2, 8(R), 9, 14(R), 18(R), 20(R), 22, 24(R)

* * * * * * * * * * * *

SUBJECT IMPRESSIONS QUESTIONNAIRE

The following sentences describe thoughts and feelings you may have had regarding the other person who participated in the experiment with you. For each of the following statement please indicate how true it is for you, using the following scale as a guide:

1	2	3	4	5	6	7
not at all true			somewhat true			very true

1. While I was interacting with this person, I was thinking about how much I enjoyed it.

2. I felt really distant to this person.

3. I did not feel at all nervous about interacting with this person.

4. I felt like I had choice about interacting with this person.

5. I would describe interacting with this person as very enjoyable.

6. I really doubt that this person and I would ever become friends.

7. I found this person very interesting.

8. I enjoyed interacting with this person very much.

9. I felt tense while interacting with this person.

10. I really feel like I could trust this person.

11. Interacting with this person was fun.

12. I felt relaxed while interacting with this person.

13. IÕd like a chance to interact more with this person.

14. I didnÕt really have a choice about interacting with this person.

15. I tried hard to have a good interaction with this person.

16. IÕd really prefer not to interact with this person in the future.

17. I was anxious while interacting with this person.

18. I thought this person was very boring.

19. I felt like I was doing what I wanted to do while I was interacting with this person.

20. I tried very hard while interacting with this person.

21. I donÕt feel like I could really trust this person.

22. I thought interacting with this person was very interesting.

23. I felt pressured while interacting with this person.

24. I think itÕs likely that this person and I could become friends.

25. I felt like I had to interact with this person.

26. I feel really close to this person.

27. I didnÕt put much energy into interacting with this person.

28. I interacted with this person because I had no choice.

29. I put some effort into interacting with this person.

Scoring information. Begin by reverse scoring items # 2, 3, 6, 12, 14, 16, 18, 21, 25, 27, and 28 by subtracting the item response from 8 and using the result as the item score for that item. Then calculate subscale scores by averaging the items scores for the items on each subscale. They are shown below. The (R) after an item number is just a reminder that the item score is the reverse of the participantÕs response on that item.

Relatedness:	2(R), 6(R), 10, 13, 16(R), 21(R), 24, 26
Interest/enjoyment:	1, 5, 7, 8, 11, 18(R), 22
Perceived choice:	4, 14(R), 19, 25(R), 28(R)
Pressure/tension:	3(R), 9, 12(R), 17, 23,
Effort:	15, 20, 27(R), 29
