# Supplementary material for: Neurotechnology-Based, Intensive, Supplementary Upper-Extremity Training for Inpatients With Subacute Stroke: Feasibility Study
Source: JMIR Serious Games. 2025 Feb 13;13:e56397. doi: 10.2196/56397 (PMC11841746; doi:10.2196/56397)
Supplement: Multimedia Appendix 2 [file games-v13-e56397-s002.pdf]

Stroke Impact Scale
VERSION 3.0

The purpose of this questionnaire is to evaluate how stroke has impacted your health and life. We want to know from **YOUR POINT OF VIEW** how stroke has affected you. We will ask you questions about impairments and disabilities caused by your stroke, as well as how stroke has affected your quality of life. Finally, we will ask you to rate how much you think you have recovered from your stroke.

Stroke Impact Scale

These questions are about the physical problems which may have occurred as a result of your stroke.

**1. In the past week, how would you rate the strength of your....**	**A lot of strength**	**Quite a bit of strength**	**Some strength**	**A little strength**	**No strength at all**
a. Arm that was most affected by your stroke?	5	4	3	2	1
b. Grip of your hand that was most affected by your stroke?	5	4	3	2	1
c. Leg that was most affected by your stroke?	5	4	3	2	1
d. Foot/ankle that was most affected by your stroke?	5	4	3	2	1

These questions are about your memory and thinking.

**2. In the past week, how difficult was it for you to...**	**Not difficult at all**	**A little difficult**	**Somewhat difficult**	**Very difficult**	**Extremely difficult**
a. Remember things that people just told you?	5	4	3	2	1
b. Remember things that happened the day before?	5	4	3	2	1
c. Remember to do things (e.g. keep scheduled appointments or take medication)?	5	4	3	2	1
d. Remember the day of the week?	5	4	3	2	1
e. Concentrate?	5	4	3	2	1
f. Think quickly?	5	4	3	2	1
g. Solve everyday problems?	5	4	3	2	1

These questions are about how you feel, about changes in your mood and about your ability to control your emotions since your stroke.

**3. In the past week, how often did you...**	**None of the time**	**A little of the time**	**Some of the time**	**Most of the time**	**All of the time**
a. Feel sad?	5	4	3	2	1
b. Feel that there is nobody you are close to?	5	4	3	2	1
c. Feel that you are a burden to others?	5	4	3	2	1
d. Feel that you have nothing to look forward to?	5	4	3	2	1
e. Blame yourself for mistakes that you made?	5	4	3	2	1
f. Enjoy things as much as ever?	5	4	3	2	1
g. Feel quite nervous?	5	4	3	2	1
h. Feel that life is worth living?	5	4	3	2	1
i. Smile and laugh at least once a day?	5	4	3	2	1

The following questions are about your ability to communicate with other people, as well as your ability to understand what you read and what you hear in a conversation.

**4. In the past week, how difficult was it to...**	**Not difficult at all**	**A little difficult**	**Somewhat difficult**	**Very difficult**	**Extremely difficult**
a. Say the name of someone who was in front of you?	5	4	3	2	1
b. Understand what was being said to you in a conversation?	5	4	3	2	1
c. Reply to questions?	5	4	3	2	1
d. Correctly name objects?	5	4	3	2	1
e. Participate in a conversation with a group of people?	5	4	3	2	1
f. Have a conversation on the telephone?	5	4	3	2	1
g. Call another person on the telephone, including selecting the correct phone number and dialing?	5	4	3	2	1

The following questions ask about activities you might do during a typical day.

**5. In the past 2 weeks, how difficult was it to...**	**Not difficult at all**	**A little difficult**	**Somewhat difficult**	**Very difficult**	**Could not do at all**
a. Cut your food with a knife and fork?	5	4	3	2	1
b. Dress the top part of your body?	5	4	3	2	1
c. Bathe yourself?	5	4	3	2	1
d. Clip your toenails?	5	4	3	2	1
e. Get to the toilet on time?	5	4	3	2	1
f. Control your bladder (not have an accident)?	5	4	3	2	1
g. Control your bowels (not have an accident)?	5	4	3	2	1
h. Do light household tasks/chores (e.g. dust, make a bed, take out garbage, do the dishes)?	5	4	3	2	1
i. Go shopping?	5	4	3	2	1
j. Do heavy household chores (e.g. vacuum, laundry or yard work)?	5	4	3	2	1

The following questions are about your ability to be mobile, at home and in the community.

**6. In the past 2 weeks, how difficult was it to...**	**Not difficult at all**	**A little difficult**	**Somewhat difficult**	**Very difficult**	**Could not do at all**
a. Stay sitting without losing your balance?	5	4	3	2	1
b. Stay standing without losing your balance?	5	4	3	2	1
c. Walk without losing your balance?	5	4	3	2	1
d. Move from a bed to a chair?	5	4	3	2	1
e. Walk one block?	5	4	3	2	1
f. Walk fast?	5	4	3	2	1
g. Climb one flight of stairs?	5	4	3	2	1
h. Climb several flights of stairs?	5	4	3	2	1
i. Get in and out of a car?	5	4	3	2	1

The following questions are about your ability to use your hand that was MOST AFFECTED by your stroke.

**7. In the past 2 weeks, how difficult was it to use your hand that was most affected by your stroke to...**	**Not difficult at all**	**A little difficult**	**Somewhat difficult**	**Very difficult**	**Could not do at all**
a. Carry heavy objects (e.g. bag of groceries)?	5	4	3	2	1
b. Turn a doorknob?	5	4	3	2	1
c. Open a can or jar?	5	4	3	2	1
d. Tie a shoe lace?	5	4	3	2	1
e. Pick up a dime?	5	4	3	2	1

The following questions are about how stroke has affected your ability to participate in the activities that you usually do, things that are meaningful to you and help you to find purpose in life.

**8. During the past 4 weeks, how much of the time have you been limited in...**	**None of the time**	**A little of the time**	**Some of the time**	**Most of the time**	**All of the time**
a. Your work (paid, voluntary or other)	5	4	3	2	1
b. Your social activities?	5	4	3	2	1
c. Quiet recreation (crafts, reading)?	5	4	3	2	1
d. Active recreation (sports, outings, travel)?	5	4	3	2	1
e. Your role as a family member and/or friend?	5	4	3	2	1
f. Your participation in spiritual or religious activities?	5	4	3	2	1
g. Your ability to control your life as you wish?	5	4	3	2	1
h. Your ability to help others?	5	4	3	2	1

9. Stroke Recovery

On a scale of 0 to 100, with 100 representing full recovery and 0 representing no recovery, how much have you recovered from your stroke?

_____ 100 Full Recovery

___

_____ 90

___

_____ 80

___

_____ 70

___

_____ 60

___

_____ 50

___

_____ 40

___

_____ 30

___

_____ 20

___

_____ 10

_______ 0 No Recovery

http://www.kumc.edu/school-of-medicine/preventive-medicine-and-public-health/research-and-community-engagement/stroke-impact-scale.html
